# Discovering pure gene-environment interactions in blood pressure genome-wide association studies data: a two-step approach incorporating new statistics

**DOI:** 10.1186/1753-6561-8-S1-S62

**Published:** 2014-06-17

**Authors:** Maggie Haitian Wang, Chien-Hsun Huang, Tian Zheng, Shaw-Hwa Lo, Inchi Hu

**Affiliations:** 1 Division of Biostatistics, School of Public Health and Primary Care, the Chinese University of Hong Kong, Shatin, New Territories, Hong Kong SAR; 2 Department of Statistics, Columbia University, 1255 Amsterdam Avenue, New York, NY 10027-5927, USA; 3 Department of ISOM, the Hong Kong University of Science and Technology, Clearwater Bay, Kowloon, Hong Kong SAR

## Abstract

Environment has long been known to play an important part in disease etiology. However, not many genome-wide association studies take environmental factors into consideration. There is also a need for new methods to identify the gene-environment interactions. In this study, we propose a 2-step approach incorporating an influence measure that capturespure gene-environment effect. We found that pure gene-age interaction has a stronger association than considering the genetic effect alone for systolic blood pressure, measured by counting the number of single-nucleotide polymorphisms (SNPs)reaching a certain significance level. We analyzed the subjects by dividing them into two age groups and found no overlap in the top identified SNPs between them. This suggested that age might have a nonlinear effect on genetic association. Furthermore, the scores of the top SNPs for the two age subgroups were about 3times those obtained when using all subjects for systolic blood pressure. In addition, the scores of the older age subgroup were much higher than those for the younger group. The results suggest that genetic effects are stronger in older age and that genetic association studies should take environmental effects into consideration, especially age.

## Background

Gene-environment interactions (G × E) have long been known to play an important role in complex disease etiology. Understanding these will reduce the bias in variable selection because of different cohort exposure to theenvironment [1]. Previous methods of studying G × E effects have mainly included candidate genes, case-only design, and family-based association studies [1,2]. These methods have made their respective assumptions in terms of biological knowledge, independence of gene and environment, and kinship information. There is an urgent need for new methods to detect gene-environment effects. With the emergence of genome-wide association studies (GWAS), data mining methods, such as generalized linear models incorporating G × E terms, are becoming popular [3,4]. We do not know,however, how much of the association identified is a result of main effects and how much is a result ofpure G × E interactions. In this study, we used a 2-step method that, first, aggressively removed main effects from both gene and environment, and then tested for the strength of pure G × E interaction. We found that, for systolic blood pressure (SBP), the pure gene-age interaction was stronger than the main effect of single-nucleotide polymorphisms (SNPs) alone. We also analyzed the genetic association separately in two age groups to test the effect of age. We found that the marker profiles were quite distinct in different age cohorts. This suggested that age might have a strong nonlinear effect on genetic association.

## Methods

### Dataset

The dataset adopted in this study was provided by Genetic Analysis Workshop 18 (GAW18), for which real phenotypes and genotypes from the San Antonio Family Studies are used. We focused on chromosome 3, which includes 62,915 SNPs. There were 142 unrelated individuals, for whom information is available on SBP, diastolic blood pressure (DBP), age, smoking status, and use of antihypertension medication. Although there were 4longitudinal measurements of the phenotypes, we considered only the first measurement, which had the fewest missing values. After removing the missing values, the data for 130 unrelated individuals were retained for further analysis.

### Detecting pure G × E effects

#### Step 1: Removal of main effect of gene and environment

For each SNP and an environmental factor, we remove their main effects on *y *by taking the residual (*res*) of projection pursuit regression (PPR) [5]. The PPR smooths the regression surface following an additive model of (nonlinear) smoothing functions (S) based on a linear combination of predictors (*α_m_*·*x*), expressed as follows:

$$y=\overset{M}{\underset{m=1}{\sum }}S_{\alpha_{m}}\left(\alpha_{m}\cdot x\right)+\epsilon$$

where $S_{\alpha_{m}}$ is the *m*^th ^smooth function of any linear combinations of *x*. Because PPR does not assume linear relation of the predictors, both nonlinear and linear effects can be removed from the residual. It is calculated using R package *ppr *in a stepwise manner by first removing the main effect of the environment, and then the main effect of the gene, without considering the interactions among them.

#### Step 2: Evaluation of interactions by an influence measure

An influence measure wasintroduced by Lo and Zheng [6] to capture the interaction effects based on partitions by a variable subset. It has been shown to be very effective in capturing joint effects, even when main effects are weak. Important SNPs were found for inflammatory bowel disease and confirmed by later experimental results [7]. This also worked in a classification algorithm that achieved the lowest error rates in predicting several cancer datasets [8].

Assuming that we have discrete explanatory variables, for a given subset of variables (either gene-gene [G × G] or G × E), a partition of the observations can be created. For example, if *x_1 _*and *x_2 _*take values of either 0 or 1, we will have a partition of four cells. If the phenotype of interest is *Y*, the influence measure takes the form

$$I=n^{-1}\underset{i}{\sum }n_{i}^{2}{\left(\bar{Y_{i}}-Y\right)}^{2}$$

where *i *runs through the partition cells, *n_i _*is the number of observations in cell *i, n *is the total number of observations, ${Ȳ}_{i}$ is the local mean of phenotypes in cell *i*, and *Ȳ *is the overall mean. When the partition contains no association information, cell mean ${Ȳ}_{i}$ should be very close to the overall mean *Ȳ*. By contrast, when a subset of variables has a joint influence on Y, the difference between ${Ȳ}_{i}$ and *Ȳ *will be large. The effect will be captured by the squared deviation and weighted by *n_i_^2^*, resulting an elevated I-score. The proposed method complements main effect methods. So one can find main effect first by using another method, and then add the interaction features back.

For each SNP and an environmental factor, the phenotype of interest (*Y*) is replaced by the residual calculated in Step 1, resulting in:

$$I=n^{-1}\underset{i}{\sum }n_{i}^{2}{\left(\bar{res_{i}}-\bar{res}\right)}^{2}$$

The significance of the I-score is evaluated by permutation on the phenotype of the data set 10^7 ^times.

#### Dichotomization of age

Smoking and medication are both discrete variables. We dichotomize age by a 2-mean clustering method (*k-means *in R). The cutoff value was found to be 55. Thus, if age is >55 years, the age is coded as 1, otherwise it is coded 0.

### Nonlinear gene-age association

Current GWAS assume that a biomarker affects disease, independent of age, so most SNPs identified in the literature are those with strong association across the whole age range. What if some genetic effect is nonlinear with age: In one's youth a group of SNPs influences the phenotype,whereas in old age some other group of SNPs takes effect? To test this hypothesis, we divided the individuals into two groups by the same 2-mean clustering threshold, at age 55 years. There were 76 subjects age ≤55 years (the younger group) and 54 subjects age >55 years (the older group). We selected the top SNPs (G effect) within each group by I-score and checked to what extentthese SNPs overlapped.

## Results

### Detecting pure gene-environment effects

#### Pure gene-age association is stronger than SNP main effect for SBP

Using the 2-step approach, the I-score of the pure interactions of G × E was calculated after the main effects of both SNP and environmental factorswere removed;*p*values were obtained by permuting the phenotype 10^7 ^times. Table 1 displays the number of SNPs, for which corresponding pure G × E interactions reached each significance level. The result for G alone appears in the last row of the table. Pure G × E interaction shows a strong association, even when the main effect has been taken away. Consider,for example, SBP: gene-age (G × age) interaction resulted in 150 SNPs with a *p *value <10^−3 ^and 29 SNPs with a *p*value <10^−4^, far more than the main genetic effect, which had only 41 SNPs with *p*value <10^−3 ^and 5 SNP with *p*value <10^−4^. Smoke and medication had no pure interaction effects with *p*value <10^−3^. For comparison purposes, the main effects of E only are also calculated, using the F-statistics of a linear regression model with all E terms included, which had a *p *value of 4.15 × 10^−13^; the main effects of all E terms on DBP gave a *p*value of 9.97 × 10^−5^.

**Table 1:** The number of SNPs reaching three levels of significance (via permutation)

Significance level reached		<10^−3^	<10^−4^	<10^−5^
G × age*	SBP	150 (0.24%)*	29 (0.46%)	1 (0.0016%)
	DBP	92 (0.15%)	11 (0.17%)	4 (0.0064%)

G^†^	SBP	41 (0.65%)	5 (0.0079%)	1 (0.0016%)
	DBP	58 (0.92%)	6 (0.0095%)	0 (0)

The percentages of the number of significant SNPs out of total number of SNPs (62,915) are shown in parentheses.

*The pure G × age interactions found by 2-step method.

^†^The main effect of G by I-score.

### Nonlinear gene-age association

#### Analysis for SBP

The subjects were divided into two groups (older than age 55 years or 55 years of age and younger) and the I-score of SNPs within each age group was calculated and ranked (Table 2). There were 3very interesting observations:

**Table 2:** Nonlinear age effect on genetic association for SBP

a. Age ≤55 years (76 observations)					
**Rank**	**SNP no**	**SNP name**	**Gene**	**I-score**	**Overall I-score**

1	14166	rs9834970	NA	77.94	20.70
2	4557	rs159154	*BRPF1*	68.90	19.03
3	12457	rs12493391	NA	68.29	34.50
4	58703	rs2239626	*DGKG*	65.98	10.36
5	269	rs12715600	NA	65.86	39.59
6	32020	rs1350790	NA	65.69	12.43
7	24555	rs10510935	NA	64.50	27.97
8	55694	rs1454149	NA	63.61	14.63
9	24613	rs4688557	NA	61.44	11.93
10	24461	rs1517931	NA	59.60	18.07

b. Age >55 years (54 observations)					

**Rank**	**SNP no**	**SNP name**	**Gene**	**I-score**	**Overall I-score**

1	49032	rs9825291	NA	172.46	63.61
2	25762	rs7427984	NA	167.36	57.98
3	36285	rs7647147	NA	150.21	93.71
4	60876	rs2669973	NA	149.84	59.60
5	1951	rs711578	*LRRN1*	148.09	76.97
6	51800	rs16851260	NA	142.42	90.44
7	49026	rs9875837	NA	139.68	55.26
8	33365	rs17176829	NA	134.32	41.67
9	62781	rs2686110	*BDH1*	134.12	54.28
10	60748	rs1016618	NA	133.97	74.78

1. There was no overlap between the top SNPs from the two age groups (the first overlap occurred at the 202nd and 92nd SNP in the two groups, respectively).

2. The I-scores of the top SNPs in age subgroups were about 3times as great as the overall I-scores calculated disregarding age (using all subjects) (see Table 2). We know that under the null hypothesis, when no association exists for a marker subset, the expected I-score is 1. The result suggests that, in this dataset, most genetic SNPs did not affect blood pressure uniformly across all age ranges. The number 1 marker rs16851260, which has an I-score of 90.44 identified by pure SNP-age interaction using 130 subjects, only ranked 6th in the subgroup of age >55 years but had a much higher I-score of 142.42. This means that this marker has a stronger genetic association in the older age group and, if calculating it using the general population, would dilute this marker's association effect.

3. Moreover, for SBP, the average I-score in the older age group is much higher than in the younger subgroup. For example, using the top 10 markers, the difference is 2.2 times. The result suggests that genetic association for SBP is much stronger in the older age group than in the youngerage group.

#### Analysis for DBP

Similar to previous results for SBP, for DBP, nonoverlapping top genetic SNPs were observed in the younger and older age groups (Table 3). The first overlapping top marker occurred at the 69th and 108th place in the two groups, respectively, which suggests that there might be a nonuniform genetic effect across age range. In addition, the association effect in older age subgroups is stronger than using all subjects, reflected by the higher I-score of the subgroup than when using all subjects. Finally, the average I-score in the older age group is much higher than in the younger group. As an example, the difference is 1.4 times for the top 10 markers. This shows that the genetic effect is slightly stronger in old age than in youth for DBP. Overall, the findings for DBP are consistent with those for SBP, but with weaker magnitude.

**Table 3:** Nonlinear age effect on genetic association for DBP

a. Age ≤55 years (76 observations)					
Rank	SNP no	SNP name	Gene	I-score	Overall I-score

1	36509	rs11706066	*SIDT1*	50.64	23.46
2	310	rs12637032	NA	50.50	23.34
3	51841	rs13094783	NA	46.00	42.74
4	24461	rs1517931	NA	45.76	18.07
5	26613	rs7620998	*EIF4E3*	44.49	11.83
6	14309	rs336597	*GOLGA4*	44.42	22.46
7	59571	rs12696583	*LPP*	44.40	9.83
8	3488	rs7628504	*GRM7*	43.66	11.35
9	38122	rs4687833	*IGSF11*	43.51	13.31
10	14166	rs9834970	NA	43.22	20.70

b. Age >55 years (54 observations)					

Rank	SNP no	SNP name	Gene	I-score	OverallI-score

1	51531	rs1996264	NA	66.64	40.85
2	51532	rs10936243	NA	66.64	40.85
3	51534	rs11918801	NA	66.64	40.85
4	51537	rs12639469	NA	66.64	40.28
5	52553	rs6801576	NA	64.79	12.85
6	40969	rs11717333	NA	62.55	24.00
7	51533	rs10513572	NA	62.14	17.60
8	62781	rs2686110	*BDH1*	62.14	54.28
9	17446	rs6799581	NA	62.03	31.33
10	40970	rs13066695	NA	59.06	16.33

## Discussion

### Considering SBP and DBP separately in GWAS

Many epidemiology studies have indicated different physiology and trend of development for SBP and DBP. It has been reported that systolic pressure is related to the elasticity of the great vessels and diastolic pressure to peripheral resistance resulting from muscle stiffness [9]. Consistent with this knowledge, the important SNPs identified for SBP and DBP in our study had few overlaps, either marginally or interactively. The results suggested that it might be better to study the two component blood pressures separatelywhen analyzing hypertension.

### Considering age group separately in GWAS

In addition to finding that pure SNP-ageinteraction was stronger than the main genetic effect, we also found, by showing that the top identified genetic SNPs were completely different between age groups,that genetic effect on blood pressure was nonlinear with respect to age.

We could estimate the probability (*p* value) of obtaining two nonoverlapping sets of top markers, under the null hypothesis that the true associated SNPs for the two age groups are identical. Suppose there are 200 true SNPs influencing SBP1 on chromosome 3, and that they are the same for both age groups. What is the probability that the two groups get complete nonoverlapping true positives (TPs). First, we need to estimate the number of TPs selected for the two age groups. This could be done by the procedure of controlling the false discovery range (FDR)[10] with *p*values obtained by permutations. The procedure says:*p_k_*≤ (*k*/*m*) q*, where *m *is the total number of tests, here *m*= 62915, *p_k _*is the *k*^th^*p*value ranked from smallest to largest, and q^* ^is the FDR. So with the permuted *p*values, we can estimate the FDR in the *k*SNPs. For the younger age group, there are 18 SNPs with *p*values ≤10^−4^, and the estimated FDR = 0.35. So the expected number of TPs = 0.65*18 ≈12 in the top 18 SNPs. For the older age group, there are 64 SNPs with *p*values ≤10^−4^, and the estimated FDR = 0.098. The number of TPs = 0.902*64 ≈ 58 in the top64 SNPs. The probability of having no younger group TP in older group TP can be calculated using a hypergeometric probability:

$$P=\frac{\left(\begin{array}{c} 200-12\\ 58 \end{array}\right)\left(\begin{array}{c} 12\\ 0 \end{array}\right)}{\left(\begin{array}{c} 200\\ 58 \end{array}\right)}=0.014$$

If the number of true markers is assumed to be 100, this probability is much more significant at 10^−5^.

Also, the strength of genetic association was much stronger in the older group than in the younger, especially for SBP. The results suggest that age has a nonlinear impact on genetic association and that the nonlinear effect of age should be considered in GWAS, perhaps by conducting studies in separate age groups. Becausethis study has a limited sample size, further research on larger numbers of subjects should be conducted.

### Pure G × E interaction-identified SNPs

The pure G × age interaction identified for SBP with *p*value reaching 10^−6^is rs6446285 on gene *BSN*. The gene is involved in the organization of the cytomatrix at the nerve terminal's active zone that regulates neurotransmitter release, and is involved in the formation of retinal photoreceptor ribbon synapses [11]. The 4SNP-age interactions reaching 10^−6 ^for DBP are all from gene *PBRM1*. Mutations at this locationhave been associated with renal cell carcinoma [12].

## Conclusions

This study demonstrated the strong G × E interactions for blood pressure. Even when main effect has been removed, pure G × E effect could be stronger than using main effect alone for SBP. The study also preliminarily explored the nonlinear age effect on genetic association and confirmed the hypothesis that some SNPs had a strong influence in a particular age range, and that the genetic effect might not be uniform across a person's lifespan. The results suggest that past GWAS might have captured only a small group of very influential SNPs that are effective regardless of age or other environmental factors. There might be a lot more SNPs, such as those shown in this study, that are "turned on" only in a particular age range and remain to be identified. These SNPs might fill in the missing heritability in the picture of GWAS.

## Competing interests

The authors declare that they have no competing interests.

## Authors' contributions

MHW carried out the statistical analysis, conceived part of the study, and drafted the manuscript. CHH processed the raw data for analysis. SHL and TZ participated in the design of statistical analysis. IH conceived the study, designed the statistical analysis, and helped to draft the manuscript. All authors have read and approved the final manuscript.

## References

[B1] Thomas D Gene-environment-wide association studies: emerging approaches Nat Rev Genet 2010 11 259 272 10.1038/nrg2764 20212493 PMC2891422

[B2] Hunter DJ Gene-environment interactions in human diseases Nat Rev Genet 2005 6 287 298 15803198 10.1038/nrg1578

[B3] Kraft P Exploiting gene-environment interaction in genome-wide association scans Ann Hum Genet 2007 71 557 558

[B4] Murcray CE Lewinger JP Gauderman WJ Gene-environment interaction in genome-wide association studies Am J Epidemiol 2009 169 2 219 226 19022827 10.1093/aje/kwn353 PMC2732981

[B5] Friedman JH Stuetzle W Projection pursuit regression J Am Stat Assoc 1981 76 817 823

[B6] Chernoff H Lo SH Zheng TA Discovering influential variables: a method of partitions Ann Appl Stat 2009 3 4 1335 1369 10.1214/09-AOAS265

[B7] Lo SH Zheng T A demonstration and findings of a statistical approach through reanalysis of inflammatory bowel disease data Proc Natl Acad Sci USA 2004 101 28 10386 10391 10.1073/pnas.0403662101 15231995 PMC478581

[B8] Wang HT Lo SH Zheng T Hu IC Interaction-based feature selection and classification for high-dimensional biological data Bioinformatics 2012 28 2834 2842 10.1093/bioinformatics/bts531 22945786 PMC3577111

[B9] Kannel WB Gordon T Schwartz MJ Systolic versus diastolic blood pressure and risk of coronary heart disease-Framingham Study Am J Cardiol 1971 27 335 337 10.1016/0002-9149(71)90428-0 5572576

[B10] Benjamini Y Hochberg Y Controlling the false discovery rate: a practical and powerful approach to multiple testing J Roy Stat Soc B Met 1995 57 1 289 300 10.2307/2346101

[B11] NCBI Gene Database http://www.ncbi.nlm.nih.gov/gene

[B12] PBRM1 gene cards http://www.genecards.org/cgi-bin/carddisp.pl?gene=PBRM1

